# Comparison of Cognitive Change after Working Memory Training and Logic and Planning Training in Healthy Older Adults

**DOI:** 10.3389/fnagi.2017.00039

**Published:** 2017-02-28

**Authors:** Vina M. Goghari, Linette Lawlor-Savage

**Affiliations:** ^1^Department of Psychology, University of Toronto, TorontoON, Canada; ^2^Department of Psychology, University of Calgary, CalgaryAB, Canada

**Keywords:** cognitive training, seniors, working memory, fluid intelligence, executive functioning

## Abstract

Recent attention has focused on the benefits of cognitive training in healthy adults. Many commercial cognitive training programs are available given the attraction of not only bettering one’s cognitive capacity, but also potentially preventing age-related declines, which is of particular interest to older adults. The issue of whether cognitive training can improve performance within cognitive domains not trained (i.e., far transfer) is controversial, with meta-analyses of cognitive training both supporting and falsifying this claim. More support is present for the near transfer (i.e., transfer in cognitive domain trained) of cognitive training; however, not in all studies. To date, no studies have compared working memory training to training higher-level processes themselves, namely logic and planning. We studied 97 healthy older adults above the age of 65. Healthy older adults completed either an 8-week web-based cognitive training program on working memory or logic and planning. An additional no-training control group completed two assessments 8-weeks apart. Participants were assessed on cognitive measures of near and far transfer, including working memory, planning, reasoning, processing speed, verbal fluency, cognitive flexibility, and creativity. Participants improved on the trained tasks from the first day to last day of training. Bayesian analyses demonstrated no near or far transfer effects after cognitive training. These results support the conclusion that performance-adaptive computerized cognitive training may not enhance cognition in healthy older adults. Our lack of findings could be due to a variety of reasons, including studying a cohort of healthy older adults that were performing near their cognitive ceiling, employing a training protocol that was not sufficient to produce a change, or that no true findings exist. Research suggests numerous study factors that can moderate the results. In addition, the role of psychological variables, such as expectations and motivation to train, are critical in understanding the effects of cognitive training.

## Introduction

Maintaining cognitive functioning is a hallmark of successful aging. Cognitively high-functioning older adults are more socially engaged, less lonely, less physically frail, and have higher overall quality-of-life ratings than cognitively lower-functioning older adults (Langlois et al., 2012; Boss et al., 2015; Halil et al., 2015). Unfortunately, cognitive proficiency is known to naturally decline with increasing age, with process cognitive abilities being particularly vulnerable (Salthouse, 2009). Process cognitive abilities refer to those which depend on moment-to-moment online cognitive processing, as opposed to drawing on previous knowledge and experience (referred to as product cognitive abilities). For example, working memory, processing speed, reasoning, and higher-level executive functions involved in planning, sequencing, coordinating, and inhibiting behaviors are process cognitive abilities, all shown to decrease in healthy aging (Salthouse et al., 2003; Salthouse, 2009). Given the rapidly aging population, discovering methods to maintain or enhance process cognitive functioning is crucial to helping older adults maintain quality of life and age well within the community.

Within the cognitive training literature, it is well-established that training a specific cognitive ability results in improvements in that task (i.e., target of practice) and generally in similar tasks (i.e., near transfer). Near transfer is defined as improvement in a task that is within the same cognitive domain as the trained task (Morrison and Chein, 2011). For example, training on a working memory task that requires one to remember a sequence of letters and block positions will result in improvements on that specific task (practice), and may also result in improvements on different working memory tasks such as mentally rearranging and recalling numbers (near transfer). However, more relevant to day-to-day functioning is whether training a particular cognitive domain transfers to improvement across other, untrained cognitive domains. This concept is termed far transfer (Morrison and Chein, 2011) and is the subject of much debate in the cognitive training literature (Morrison and Chein, 2011; Shipstead et al., 2012; Melby-Lervåg and Hulme, 2013).

Similarly, in healthy older adults, near transfer after cognitive training is largely reliable; however, far transfer is more contested. Improving fluid intelligence is typically the main far transfer target for studies of working memory training. Therefore, we aimed to improve fluid intelligence via near transfer by training higher-level skills themselves, namely logic and planning. We compare the effects of directly training these higher-level skills to that of training working memory.

Although numerous forms of cognitive training exist, working memory training has garnered the most attention in healthy adults. The hypothesized objective of working memory training is to enhance an individual’s core ability to temporarily store and process information. Working memory training aims to increase the core capacity and processing efficiency of working memory, factors which are important for day-to-day cognitively demanding activities such as language, reasoning, problem solving, reading comprehension, and more general aspects of knowledge-based and fluid intelligence (Cowan, 2010; Thompson et al., 2013). Relative to younger adults, older adults seem to have lower storage capacity and are more susceptible to distraction (Gazzaley et al., 2005; Basak and Zelinski, 2013); therefore, working memory training is a reasonable intervention target when the goal is to enhance broader cognitive processes. In a meta-analysis specific to working memory training interventions, in healthy adults over 60 years of age, Karbach and Verhaeghen (2014) reported large and significant near transfer after working memory training, and significant albeit smaller effects on far transfer, suggesting this intervention holds promise.

Another potential approach to cognitive training is to more directly target higher-order executive functioning processes. Executive functioning is a broad term that describes higher-order cognition, including reasoning, planning, and cognitive flexibility. Working memory is also often included as a domain of executive functioning. In a latent variable analysis, task-switching had a strong and significant relationship with performance-based instrumental activities of daily living in a sample of healthy older adults aged 60–90 years old, indicating that executive functioning may impact everyday activities in the elderly (Vaughan and Giovanello, 2010). Furthermore, Karbach and Verhaeghen’s (2014) meta-analysis revealed significant and robust near transfer for executive functioning training (not including working memory specifically), although less robust far transfer, after training which targeted attention, inhibition, task-switching, and dual-task performance. Hence, training of executive functioning may be a more direct route (near transfer to other executive processing skills), relative to working memory training (far transfer to other executive processing skills), to improve a wider range of cognitive abilities.

Logic and planning is an aspect of executive functioning involved in decision making and problem solving. Only one study to date has included a planning intervention with older adults. Zinke et al. (2014) trained 80 older adults (aged 65–95 years) on working memory and an executive control tower task which required planning. In addition to finding near transfer to simple visual and auditory span tasks and a planning task, far transfer to a fluid intelligence task was reported, although Redick (2015) refuted this conclusion. Furthermore, since the training program included both a planning task and working memory tasks, it is impossible to tease apart the effects of working memory training from that of planning training.

To our knowledge, no prior studies have examined the impact of specifically training higher-level logic and planning and compared it to the most widely used single domain targeted training, working memory training, in healthy, community dwelling, older adults (>65). The primary goal of the study was to identify whether working memory training versus logic and planning training differentially impacts cognitive performance on a variety of near and far transfer tasks. Furthermore, we aimed to discover whether either type of training benefited healthy older adults relative to usual activities (i.e., a no-contact passive control condition). Given previous findings regarding the beneficial impacts of working memory training for healthy older adults, we expected that our working memory trainees would demonstrate improvements in at least the trained domain of working memory (i.e., near transfer to working memory tasks), and potentially in measures of far transfer (i.e., executive functioning, reasoning, processing speed, and creativity tasks). We also anticipated that our logic and planning trainees would demonstrate improvements in tasks tapping logic and planning (i.e., near transfer to planning and non-verbal reasoning), and potentially in far transfer tasks (i.e., working memory, processing speed, verbal fluency, cognitive flexibility, and creativity). Although, it can be argued that working memory, processing speed, and cognitive flexibility may not be far transfer tasks for logic and planning training, we grouped these tasks under far transfer, as those cognitive domains were not specifically targeted or trained. Last, we anticipated that both training groups would demonstrate these improvements relative to the passive control group.

## Materials and Methods

### Participants

Healthy adults over the age of 65 were recruited from the community in Calgary, AB, Canada. Informed consent was obtained from each participant online, as well as in-person. Study procedures were approved by and carried out in accordance with the University of Calgary Conjoint Faculties Research Ethics Board. Potential participants completed an online screening questionnaire to assess eligibility. Exclusion criteria were age less than 65, lack of English proficiency, history of head trauma, brain fever, self-reported neurological or psychiatric illness, dementia, or altered consciousness, use of benzodiazepines or illicit drugs in past 3 months, current visual, auditory, or motor impairment, cardiovascular condition, respiratory problems, and a score less than 27 on the Mini Mental State Examination (MMSE). Last, all participants needed access to a high speed internet connection.

Individuals meeting inclusion criteria were then invited to attend an in-person, individual cognitive assessment. Prior to the assessment, participants completed online questionnaires assessing demographics, mood, physical activity, and sleep quality. Study eligibility was further confirmed in-person. After the assessment, participants were quasi-randomized (accounting for sex distribution) to one of three groups: working memory training, logic and planning training, or a passive (i.e., no-training) control group. A research assistant introduced participants in the two training groups to the BrainGymmer website and games. Both training groups were instructed to train, at a time and location of their convenience, for approximately 30 min per day, 5 days a week, for 8-weeks, totaling 20 h of training. Adherence to training was monitored weekly and phone calls or emails followed if participants deviated from the protocol. All groups completed a second assessment after 8 weeks. Participants in the training groups received no remuneration, but were entered into one of several draws held throughout the study. We did not pay participants in the two training groups, as research suggests remuneration for training reduces participants’ intrinsic motivation to train, which is one necessary factor for training to work (Au et al., 2015). To provide an incentive to the passive control group to participate, this group was paid $25 per assessment.

### Training Programs

The working memory and logic and planning training games were provided by BrainGymmer^1^. All games were adaptive to ensure games remained both engaging and challenging, but not frustrating. The criteria for adaptation were unique to each game and based on error thresholds. For example, for the n-back game, if participants achieved greater than 80% accuracy on 15 trials, they moved up in difficulty level, and if they achieved less than 80% accuracy, they moved down in difficulty. Participants began each training session at the lowest level of difficulty. Three games were chosen in each domain to reduce boredom, which was a potential problem in a previous study (Lawlor-Savage and Goghari, 2016), and to provide more comprehensive training within the cognitive domain.

#### Working Memory Games

The three games in this domain primarily targeted maintenance and manipulation of information. In the Multi-Memory game, a square grid was presented and different tiles were placed on the grid. Participants had to remember the placement of the tiles, which then disappeared and were replaced by a distractor pattern. For each trial, participants had to recreate the original pattern of tiles. The size of the square grid and number of tiles changed as a function of performance. In the Moving Memory game, pairs of cards were shown with the same image, but with different numbers at the bottom. The cards were then flipped and scrambled with only the number on the card visible. For each trial, participants had to pick the two cards with the same image, until no pairs of cards remained. The number of pairs to be remembered changed as a function of performance. Last, in the *N*-back game, a pattern was shown facing up and was then flipped over, so the pattern was no longer visible. Then a different number of cards appeared and participants had to respond if the card presented was the same as “n” back. The number of cards to be remembered changed as a function of performance.

To investigate the relationship between the tasks, day 1 scores from each task were correlated: specifically, highest n-back achieved for the n-back task, and mean score and difficulty level for Multi-Memory and Moving Memory. Day 1 scores among the three working memory games were correlated (*r*’s = 0.32–0.51, *p*’s = 0.001–0.058).

#### Logic and Planning Games

The three games in this domain primarily targeted planning, reasoning, and problem solving abilities. In the Square Logic game, a grid of numbered squares was presented. The objective was to stack the squares using the rule that squares can only be stacked onto squares that are one point higher or lower in value. The number of squares to stack changed as a function of performance. In the Out of Order game, a series of squares were presented, each with different shapes, patterns within the shape, color, and number of shapes. The objective of this game was to rearrange the squares so that each square matched at least one characteristic of the square adjacent to it. The number of squares to arrange changed as a function of performance. Last, in the Patterned Logic game, a pattern with missing pieces was presented. Participants had to choose the correct piece to complete the pattern. Pattern complexity changed as a function of performance.

Correlations among the games were conducted based on day 1 mean score and difficulty level. The Square Logic game mean score was associated with Pattern Logic game mean score (*r* = 0.39, *p* = 0.03). Also, the Square Logic game difficulty level achieved was correlated with the Patterned Logic game mean score and difficulty level (*r*’s = 0.48–0.56, *p*’s = 0.001–0.007). Furthermore, the Square Logic game difficulty level and Out of Order game difficulty level achieved were inversely associated (*r* = -0.36, *p* = 0.05). Last, Patterned Logic game difficulty level and Out of Order game difficulty level were inversely associated (*p* = -0.47, *p* = 0.008). This suggests the Out of Order game tapped different executive functioning processes than the other two games.

### Baseline Measures

At baseline, participants completed an online self-report demographics questionnaire and inventories of state characteristics (mood, physical activity, sleep quality) commonly known to impact cognitive performance. Mood was measured with the Beck Depression Inventory-II (BDI-II; Beck et al., 1996) and the Beck Anxiety Inventory (BAI; Beck and Steer, 1990). The BDI-II is a 21-item self-report of depressive symptoms and has well-established reliability and validity in broad populations, including healthy older adults (Dozois et al., 1998; Segal et al., 2008). The BAI is a 21-item scale measuring severity of anxiety symptoms, and has demonstrated excellent reliability and validity in healthy older adults (Kabacoff et al., 1997). Physical Activity was measured with the Rapid Assessment of Physical Activity (RAPA; Topolski et al., 2006), a 9-item self-report inventory of physical strength, flexibility, and physical activity intensity for adults over age 50 years. Sleep was assessed with the Pittsburgh Sleep Quality Index (PSQI; Buysse et al., 1989), a self-report measure of subjective sleep quality and daytime dysfunction. The PSQI has demonstrated high test-retest reliability, sensitivity, and specificity in detecting sleep difficulties in clinical and non-clinical populations (Buysse et al., 1989). General cognitive ability was estimated with the Wechsler Abbreviated Scale of Intelligence-Second Edition (WASI-II), a short yet well-validated measure of overall cognitive performance (Wechsler, 2011).

### Outcome Measures

At baseline and post-training, training group participants provided motivation ratings. Specifically, participants responded to the question, “How motivated would you say you are/were to complete the cognitive training component of this study?” by marking a 7-point scale ranging from no motivation to substantial motivation.

At baseline and post-training, all participants underwent testing of working memory, processing speed, executive functioning (logic and planning, verbal fluency, cognitive flexibility), creativity, and reasoning. Measures were chosen to tap a variety of near and far transfer cognitive processes, for their use in previous investigations of working memory training, their sensitivity to age-related differences in cognitive performance, and their reliability, validity, and utility in the cognitive assessment of healthy older adults. Cognitive tasks were grouped by cognitive domain based on conceptual relationships among specific measures.

#### Working Memory (Near Transfer for Working Memory Training; Far Transfer for Logic and Planning Training)

Working memory was assessed with the Digit Span subtest of the Wechsler Adult Intelligence Scale-IV (WAIS-IV; Wechsler, 2008a). Participants repeated a series of verbally presented digits verbatim, backward, and in ascending order. Digit Span total raw scores were used.

Working memory was also measured with the Automated Operation Span (Aospan) task (Unsworth et al., 2005). Participants solved mathematical operations presented on a computer screen while remembering a sequence of letters. This task is highly correlated with other measures of working memory (Conway et al., 2002; Unsworth et al., 2005) and recruits updating processes of working memory.

#### Planning (Near Transfer for Logic and Planning Training; Far Transfer for Working Memory Training)

The Tower Test (TT) was utilized to examine spatial planning, rule learning, and inhibition of impulsive responding (Delis et al., 2001).

#### Non-verbal Reasoning (Near Transfer for Logic and Planning Training; Far Transfer for Working Memory Training)

The Raven’s Advanced Progressive Matrices (RAPM; NCS Pearson Inc., 2007) is a reliable and well-validated measure of non-verbal reasoning. Although others (e.g., Colom and Garcia-Lopez, 2003; Jaeggi et al., 2010; Schweizer et al., 2011; Stephenson and Halpern, 2013; Lawlor-Savage and Goghari, 2016) have utilized RAPM as a fluid intelligence task, we consider fluid intelligence a broader construct which encompasses matrix and non-matrix based tasks. We therefore refer to the RAPM as a non-verbal reasoning task, although in line with the literature, we also discuss the task in the context of fluid intelligence. Participants examined a picture or series of images arranged in a pattern with one piece missing, and selected the best of eight possible images to complete the pattern. The test was administered in two sets: a 5-min long 12-item practice set of increasing difficulty which ensured participants understood the task and acted as a screener for low ability, and a 40-min long 36-item test set of increasing difficulty in which the raw score (total number correct) was used as an outcome measure of reasoning. The RAPM has demonstrated convergent validity with more general measures of critical thinking and achievement and high internal consistency in adults (0.85; NCS Pearson Inc., 2007).

#### Processing Speed (Far Transfer for Working Memory and Logic and Planning Training)

Processing speed was measured using multiple tasks. Raw scores from the Symbol Search subtest of the WAIS-IV (Wechsler, 2008a) were used, in which visual stimuli were presented and participants quickly responded by identifying symbols that matched other symbols. The Symbol Search task has been described as “as pure a test as possible of information-processing speed” (Wechsler, 2008b).

Processing speed was also assessed with Trail Making Test (TMT) Items 1, 2, and 3 and Color-Word Interference Test (CWIT) Items 1 and 2, all of which are subtests of the Delis Kaplan Executive Function System (D-KEFS; Delis et al., 2001). The TMT is a visual-motor sequencing task in which the first subtest (TMT 1) measures visual scanning speed, and the next two subtests (TMT 2 and TMT 3) assess sequencing speed. The CWIT is a 4-item executive functioning task of which the first 2-items assess word reading speed (CWIT 1) and color naming speed (CWIT 2).

#### Verbal Fluency (Far Transfer for Working Memory and Logic and Planning Training)

From the verbal fluency subtest of the DKEFS, we utilized the letter fluency task (LF) to measure speeded verbal generation of words belonging to a particular phonemic category.

#### Cognitive Flexibility (Far Transfer for Working Memory and Logic and Planning Training)

Cognitive flexibility was assessed with four tasks from the DKEFS. Specifically, we used Color-Word Interference Test Item 3 (CWIT 3) which measures inhibition, and Color-Word Interference Test Item 4 (CWIT 4) which measures task-switching performance. Trail Making Test 4 (TMT 4) and Design Fluency Test 3 (DF 3) were also included to assess task-switching and inhibition.

#### Creativity (Far Transfer for Working Memory and Logic and Planning Training)

The first 2-items from the Design Fluency (DF) subtest of the DKEFS were used (DF 1 and DF 2) to assess initiation of problem-solving behavior, visual pattern generativity, creativity in drawing new designs, and inhibition of previously drawn responses.

### Statistical Analysis

To analyze the demographic, mood, sleep, physical activity, and baseline cognitive data, analyses of variance (ANOVAs) and chi-squared tests were conducted. To analyze the cognitive training data, correlations and Bayesian Repeated Measures Analyses of Variance (Bayes RM-ANOVAs) were conducted using the JASP statistics package, version 0.7, available online at https://jasp-stats.org/ (Love et al., 2015). Participants who trained for a minimum of 10 h were included in the study.

Within conceptually related cognitive domains, correlations (two-tailed) were conducted among baseline cognitive outcome measures. Based on moderate to strong correlations among some outcome measures within cognitive domains, composites were created by adding z-scores of tasks which significantly correlated (α < 0.05) within a domain. Specifically, within the working memory domain, Digit Span Total scores and Aospan scores were not significantly correlated so were analyzed individually. A processing speed composite was created using TMT 1, TMT 2, TMT 3, CWIT 1, and CWIT 2 scores, whereas Symbol Search scores were analyzed independently. A cognitive flexibility composite was created from CWIT 3 and CWIT 4 scores, and remaining tasks within the cognitive flexibility domain were analyzed separately. A DF composite was created using both tasks within that domain (DF 1 and DF 2). The logic and planning, verbal fluency, and reasoning domains were composed of single tasks analyzed individually.

Bayesian Repeated Measures Analyses of Variance were utilized to compare group differences across time for the motivation data and each cognitive composite or individual task. For the cognitive data, first, a Bayes RM-ANOVA including all three groups was conducted. If that Bayes RM-ANOVA was significant, we followed up to investigate if the training groups improved compared to the passive control group, which assessed test-retest fluctuations. If this was significant, we followed up by comparing the two training groups.

The JASP statistical analysis program generates Bayes factors using default prior probabilities; however, rather than producing a probability estimate in support of the null hypothesis based on an arbitrarily determined cut-off of statistical significance, the Bayes approach compares likelihood estimates of the obtained data occurring under the null (01) versus alternative (10) hypothesis. Advantages and specific procedures of the Bayesian RM-ANOVA approach, including the use of default priors, are extensively discussed elsewhere (e.g., Masson, 2011; Rouder et al., 2012; Jarosz and Wiley, 2014). To allow for clear interpretation, we followed the process utilized in a working memory training study by Sprenger et al. (2013) which states that *BF*_01_ < 0.33 provides support for the alternative (i.e., 3:1 probability in favor of the alternative), and *BF*_01_ < 0.05 indicates strong evidence for the alternative hypothesis (i.e., 20:1 probability in favor of the alternative). Conversely, *BF*_01_ > 3 indicates support for the null hypothesis (3:1 probability in favor of the null) and *BF*_01_ > 5 indicates strong support for the null (20:1 probability in favor of the null).

## Results

### Participant Characteristics

**FIGURE 1:**
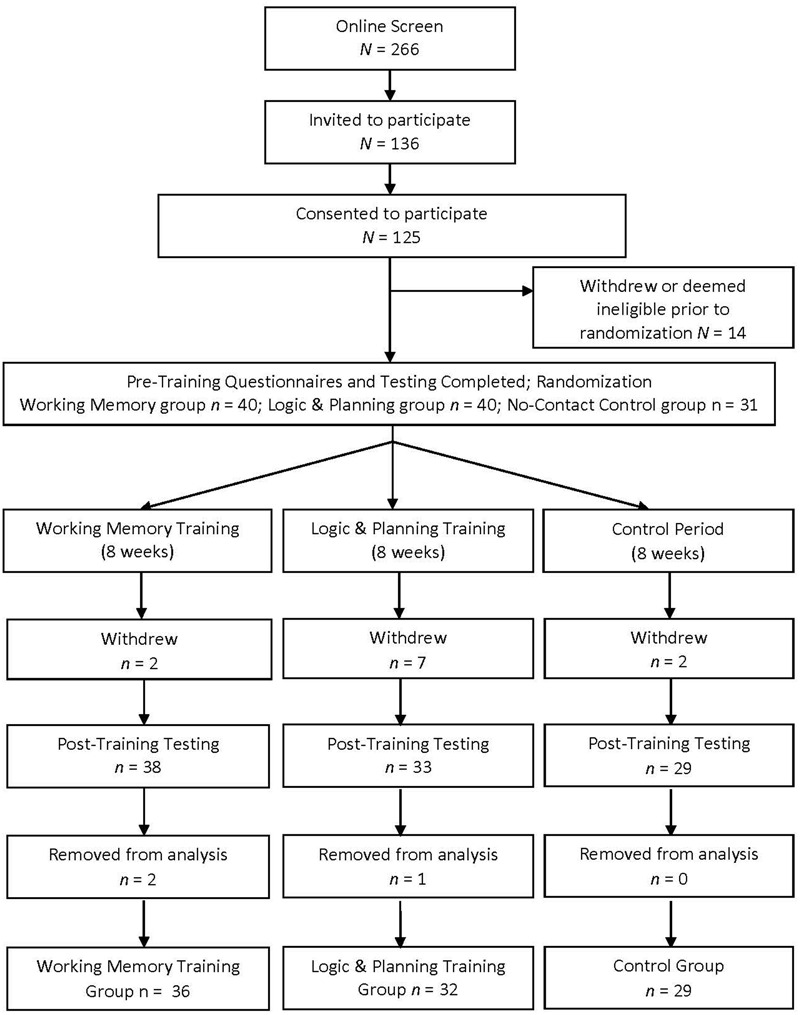
**Flow chart of study design**.

Screening, eligibility, consent, and completion rates for the working memory training, logic and planning training, and no-training control groups are presented in **Figure 1**. Of the 125 participants who initially consented, 14 participants withdrew or were deemed ineligible prior to randomization, 11 withdrew during the study (primary reasons were disliking the training, or experiencing a health difficulty), and data from three participants were removed prior to analysis due to low training dosage. Low dosage was set at completing less than 50% of the training (i.e., less than 10 h). Studies in young healthy adults suggest effects with as little as 6 h of training; however, we set our minimum hours to be higher given our older adult population (Jaeggi et al., 2008). Final analysis included 36 working memory trainees, 32 logic and planning trainees, and 29 passive control participants.

Participant characteristics are presented in **Table 1**. Participants did not differ for age [*F*(2,94) = 0.13, *p* = 0.88], sex [*X*^2^(2) = 0.25, *p* = 0.88], or education level completed [*F*(2,94) = 0.08, *p* = 0.99]. In terms of mood, participants had similar levels of self-reported depression [*F*(2,94) = 0.999, *p* = 0.37] and anxiety [*F*(2,94) = 0.83, *p* = 0.44]. Groups also had similar sleep quality [*F*(2,94) = 0.21, *p* = 0.81] and physical activity scores (*F*’s = 0.09–1.86, *p*’s = 0.16–0.91).

**Table 1:** Demographics, mood, sleep, physical activity, cognition, and training characteristics.

	Working memory training	Logic and planning training	Passive control	*p*
**Demographics**				
N	36	32	29	
Age	70.39 (4.54)	70.81 (4.98)	70.24 (4.48)	*p* = 0.88
Range	65–86	84–65	65–78	
Sex (% female)	64	69	69	*p* = 0.88
Ethnicity (% Caucasian: Asian: Other)	94: 6: 0	88: 13: 0	86: 7: 7	
Marital status (% coupled)	72	69	62	
Education (years completed)	15.43 (3.48)	15.44 (2.86)	15.52 (2.86)	*p* = 0.99
Range	7–23	10–21	9–22	
Employment (% retired)	86	81	83	
Income (% <$50,000: $50,000–$95,000: >$95,000)	31: 47: 22	55: 23: 23	32: 50: 18	
**Mood, Sleep, Physical Activity**				
Beck Depression Inventory	5.61 (6.55)	3.66 (4.29)	4.97 (6.12)	*p* = 0.37
Range	0–24	0–18	0–26	
Beck Anxiety Inventory	3.33 (4.42)	2.25 (3.22)	2.48 (3.00)	*p* = 0.44
Range	0–18	0–15	0–10	
PSQI Total	4.86 (3.03)	4.47 (3.07)	4.41 (3.09)	*p* = 0.81
Range	2–12	0–12	0–15	
RAPA Aerobics	4.83 (1.75)	5.56 (1.27)	5.14 (1.60)	*p* = 0.16
Range	0–7	4–7	2–7	
RAPA Strength	1.56 (1.30)	1.69 (1.26)	1.62 (1.24)	*p* = 0.91
Range	0–3	0–3	0–3	
**Cognition**				
MMSE	28.89 (0.95)	28.67 (1.00)	29.03 (0.94)	*p* = 0.34
Range	27–30	27–30	27–30	
WASI-II 4-item composite	111.75 (13.24)	113.94 (10.28)	112.52 (11.88)	*p* = 0.75
Range	66–133	91–135	96–148	
**Cognitive Training**				
Training time (hours)	19.01 (2.14)	19.44 (2.42)		*p* = 0.43
Range	14.23–22.68	12.32–24.87		

*PSQI, Pittsburgh Sleep Quality Index; RAPA, Rapid Assessment of Physical Activity; MMSE, Mini Mental State Examination; WASI-II = Wechsler Abbreviated Scale of Intelligence, Second Edition.*

Importantly, groups had similar MMSE scores [*F*(2,92) = 1.098, *p* = 0.34] and WASI-II full scale IQ estimates [*F*(2,94) = 0.29, *p* = 0.75]. Groups did not differ on any individual cognitive assessment task, or composite of tasks, at baseline.

### Motivation

At baseline, the null hypothesis suggesting groups were equally motivated to complete the training was supported, *BF*_01_ = 3.34. Bayes RM-ANOVA was conducted to identify group by time differences in self-reported motivation regarding training. The results demonstrated an inconclusive effect of time, *BF*_01_ = 1.55, indicating either hypothesis to be equally as likely. Importantly, for the interaction effect, evidence emerged for the null hypothesis, indicating groups were equally motivated over time, *BF*_01_ = 8.50.

### Training

Participants completed the cognitive pre-assessment and cognitive post-assessment close to beginning and ending their training [mean = 1.25 days before training, *SD* = 1.72; *F*(1,66) = 0.32, *p* = 0.58; mean = 4.18 days after training, *SD* = 5.31; *F*(1,66) = 1.23, *p* = 0.26]. Analysis of the weekly training logs demonstrated that participants practiced each of the three games for approximately 10 min each session, totalling approximately 30 min a session. The working memory and logic and planning training groups trained for a similar number of hours [*F*(1,66) = 0.62, *p* = 0.43]. Importantly, participants within each training group attained a higher level of difficulty on every game played after training (*p* < 0.001).

### Outcome Analysis by Cognitive Domain

Means, standard deviations, and Bayes factors indicating support for the null versus alternative hypotheses are presented in **Table 2**.

**Table 2:** Means and Standard Deviations before (T1) and after (T2) training period, and Bayes factors^1^ of time and interaction effects.

Domain	Task	WMT T1	WMT T2	LPT T1	LPT T2	PC T1	PC T2	Time	Group × Time
		mean (*SD*)	mean (*SD*)	mean (*SD*)	mean (*SD*)	mean (*SD*)	mean (*SD*)	(*BF*_01_)	(*BF*_01_)
Motivation		5.57 (1.37)	5.91 (1.63)	5.91 (1.63)	5.72 (1.49)	-	-	1.55	8.50
Working memory	Aospan	24.72 (14.06)	29.36 (13.92)	26.45 (14.56)	31.53 (18.12)	26.41 (15.48)	32.31 (16.36)	0.01	0.43
	DST	27.83 (4.66)	28.67 (4.67)	27.47 (6.51)	28.88 (5.93)	26.32 (5.04)	28.41 (4.98)	4.30	116.25
Planning	TT	16.34 (3.50)	18.25 (2.49)	16.69 (4.88)	18.28 (4.63)	16.17 (5.09)	18.97 (3.52)	<0.001	0.07
Reasoning	RAPM	8.22 (2.81)	8.33 (2.92)	8.28 (2.98)	8.55 (2.73)	7.41 (1.90)	8.21 (2.73)	3.72	94.92
Processing speed	Composite	-0.35 (3.23)	-0.40 (3.56)	-0.09 (4.58)	-0.16 (4.26)	0.52 (2.55)	0.67 (3.16)	6.48	161.83
	SS	28.11 (5.50)	29.56 (6.73)	29.78 (6.63)	29.97 (7.73)	26.69 (5.93)	27.45 (5.57)	1.5	14.88
Verbal fluency	LF	40.06 (10.82)	43.34 (10.85)	39.75 (10.20)	43.25 (11.79)	38.79 (10.94)	43.83 (13.89)	0.002	0.07
Flexibility	Composite	-0.05 (1.70)	-0.15 (1.88)	-0.01 (2.01)	0.05 (1.83)	0.08 (1.70)	0.14 (1.69)	6.44	142.79
	DF 3	7.31 (2.14)	7.92 (2.38)	8.47 (2.87)	9.00 (2.34)	7.14 (2.34)	8.08 (2.47)	1.23	2.98
	TMT 4	90.26 (26.54)	81.81 (27.65)	86.22 (40.30)	82.88 (41.49)	92.31 (37.94)	87.10 (29.39)	1.58	39.40
Creativity	Composite	-0.25 (1.97)	0.09 (1.77)	0.45 (2.09)	0.33 (2.14)	-0.20 (1.50)	-0.14 (1.68)	6.58	6.58

*Aospan, Automated Operation Span task; DST, Digit Span Total; SS, Symbol Search; LF, Letter Fluency; DF3, Design Fluency Condition 3; TMT4, Trail-Making Task Condition 4; TT, Tower Task; RAPM, Raven’s Advanced Progressive Matrices. Composite scores were created by summing z-scores of individual outcome tasks. Processing speed composite was composed of TMT Conditions 1, 2, 3 and Color-Word Interference Task (CWIT) Conditions 1 and 2; cognitive flexibility composite was composed of CWIT Conditions 3 and 4; Creativity composite was composed of Design Fluency (DF) Conditions 1 and 2. ^*1*^Bayes factors assessing the null hypothesis (*BF*_*01*_) < 0.33 indicates support for the alternative hypothesis (i.e., 3:1 probability in favor of the alternative), and *BF*_*01*_ < 0.05 indicates strong evidence for the alternative hypothesis (i.e., 20:1 probability in favor of the alternative). Conversely, *BF*_*01*_ > 3 indicates support for the null hypothesis (3:1 probability in favor of the null) and *BF*_*01*_ > 5 indicates strong evidence for the null (20:1 probability in favor of the null). Values between 0.33 and 3 suggest only weak support, and values close to 1 are not informative.*

#### Working Memory

Within the working memory domain, Aospan and Digit Span Total scores were not significantly correlated (*r* = 0.04, *p* = 0.73); therefore, these outcome scores were analyzed separately. Bayesian RM-ANOVA of Aospan resulted in evidence for an effect of time, *BF*_01_ = 0.01 although not for group, *BF*_01_ = 3.80. The presence of a group by time interaction was only weakly supported, *BF*_01_ = 0.43; given the weak support, we did not follow-up on this finding. Bayes RM-ANOVA of Digit Span Total scores (Digit Span Forward + Digit Span Backward + Digit Span Sequencing raw scores) provided evidence for the null hypothesis for the effect of time, *BF*_01_ = 4.30, group *BF*_01_ = 6.80, and for the interaction between group and time, *BF*_01_ = 116.25.

#### Planning

The TT assessed visual-spatial planning. For this task, evidence was provided for an effect of time, *BF*_01_ < 0.001 although not for group *BF*_01_ = 8.56, with support for a group by time interaction, *BF*_01_ = 0.07. Follow-up Bayesian ANOVAs comparing the working memory training group to the passive control group provided evidence for the alternative hypothesis regarding an effect of time, *BF_01_*< 0.001, and support for an interaction *BF*_01_ = 0.25. However, further examination of the interaction revealed that it is not a meaningful training-related outcome, as the control group changed more (mean improvement = 2.79, *SD* = 4.31) than the working memory training group (mean improvement = 1.91, *SD* = 3.23), suggesting the effects were by chance. Comparison of the logic and planning training group to the passive control group revealed evidence for an effect of time, *BF*_01_ = 0.01, with higher scores after training, and support for a group by time interaction, *BF*_01_ = 0.11. However, further examination of this interaction also suggests non-meaningful results given that the control group changed more (mean improvement = 2.79, *SD* = 4.31) than the logic and planning group (mean improvement = 1.59, *SD* = 4.53).

#### Non-verbal Reasoning

The RAPM task was used to measure reasoning. Data supported the null hypothesis for an effect of time, *BF*_01_ = 3.72, and provided evidence for the null hypothesis for the effect of group, *BF*_01_ = 6.68, and the group by time interaction, *BF*_01_ = 94.92.

#### Processing Speed

Within the processing speed domain, outcome scores among TMT Conditions 1, 2, and 3, and CWIT Conditions 1 and 2 were positively correlated (*r*’s = 0.21–0.59, *p*’s < 0.001–0.04) and were therefore analyzed as a composite. However, Symbol Search scores were negatively correlated with the other processing speed outcomes (*r*’s = -0.23 to -0.49, *p*’s < 0.001–0.03) and were therefore analyzed individually. For the processing speed composite, the data indicated evidence for the null hypothesis regarding an effect of time, *BF*_01_ = 6.48, and supported the null regarding the effect of group, *BF*_01_ = 3.62. For the interaction, the data provided evidence for the null hypothesis, *BF*_01_ = 161.83. For Symbol Search, neither hypothesis was supported regarding the effect of time, *BF*_01_ = 1.5 or group, *BF*_01_ = 1.13. The data provided evidence for the null interaction hypothesis, *BF*_01_ = 14.88.

#### Verbal Fluency

The verbal fluency task data revealed evidence for an effect of time, *BF*_01_ = 0.002, and an interaction between group and time, *BF*_01_ = 0.07. Data supported the null hypothesis regarding the group effect, *BF*_01_ = 5.21. Follow-up Bayesian RM-ANOVA comparing the working memory group to the control group provided evidence for the effect of time, *BF*_01_ = 0.01, although not for group, *BF*_01_ = 2.71, and support for the group by time interaction, *BF*_01_ = 0.08. However, further examination of the interaction revealed that it is not a meaningful training-related outcome, as the control group changed more (mean improvement = 5.03, *SD* = 8.15) than the working memory training group (mean improvement = 3.25, *SD* = 8.71), suggesting the effects were due to chance. Bayesian RM-ANOVA comparing the logic and planning group to the control group revealed evidence for the effect of time, *BF*_01_ = 0.02, although not for group, *BF*_01_ = 2.81, and only weak support for the interaction, *BF*_01_ = 0.12; therefore, no follow-up was conducted.

#### Cognitive Flexibility

Cognitive flexibility represents processes reliant on inhibition and task-switching. CWIT3 and CWIT4 were significantly correlated (*r* = 0.60, *p* < 0.001); however, TMT4 and DF3 were not correlated with each other (*r* = -0.16, *p* = 0.11). Further, TMT4 was not correlated with CWIT3 (*r* = 0.11, *p* = 0.27) or CWIT4 (*r* = 0.11, *p* = 0.27) and DF3 was negatively correlated with both CWIT3 (*r* = -0.30, *p* = < 0.01) and CWIT4 (*r* = 0.26, *p* = 0.01) so TMT4 and DF3 were analyzed independently. For the CWIT Conditions 3 and 4 composite, evidence favored the null for an effect of time, *BF*_01_ = 6.44, group, *BF*_01_ = 2.57, and the group by time interaction, *BF*_01_ = 142.79. For DF Condition 3, neither hypothesis was clearly supported for the effect of time, *BF*_01_ = 1.23, the null hypothesis was weakly supported for the group by time interaction, *BF*_01_ = 2.98, and a main effect of group was supported, *BF*_01_ = 0.33. Follow-up of this group effect revealed weak support for the null hypothesis when comparing the working memory and control groups, *BF*_01_ = 2.50, support for the alternative when comparing the logic and planning to the control group, *BF*_01_ = 0.21, with the logic and planning group generally having higher means than the control group, regardless of time-point, and support for the null when comparing the working memory and logic and planning groups, *BF*_01_ = 0.50. For the TMT 4 task, neither hypothesis was adequately supported for the effect of time, *BF*_01_ = 1.58, and evidence was for the null regarding the effect of group, *BF*_01_ = 7.43, and the group by time interaction, *BF*_01_ = 39.40.

#### Creativity

Creativity tasks were DF conditions 1 and 2, both which require drawing new images with the main restriction being to not repeat drawings. These two tasks were significantly correlated (*r* = 0.80, *p* < 0.001), so were analyzed as a creativity composite. Evidence was for the null model for both the effect of time, *BF*_01_ = 6.58, and the group by time interaction, *BF*_01_ = 43.45. The data weakly supported the null for the effect of group, *BF*_01_ = 2.09.

## Discussion

Although some level of cognitive decline is a natural part of aging, slowing, preventing, or ameliorating cognitive decline is a key goal of healthy living. In this study, we investigated near and far transfer of two active conditions, working memory training and logic and planning training, to a passive control group, in healthy community dwelling seniors (age 65 plus). Working memory training was chosen as it is the most widely studied process-specific training and is conceptually related to fluid intelligence (the most common far transfer goal). As a comparison condition, we also, for the first time, trained logic and planning to investigate its potential to transfer to fluid intelligence as near transfer. Given the positive benefits of greater fluid intelligence to healthy living, this is an important conceptual question. In this sample of 97 healthy older adults, we found only evidence for improvements on the trained tasks, and none for near or far transfer after cognitive training.

After training for an average of 19 h, we found both groups performed substantially better on all the training tasks compared from the first day of training to the last day of training. However, surprisingly, we found no evidence for near transfer. Neither maintenance nor manipulation, as measured by the Digit Span task, or working memory capacity, as measured by the Aospan task, improved relative to the control group. Similarly, the logic and planning training group did not improve on the Tower task, which measures planning. Furthermore, scores on the Raven’s Progressive Matrices task, a common measure of reasoning, did not improve after logic and planning training.

In addition to near transfer, we also investigated far transfer for both training conditions. Far transfer for working memory training was assessed by measures of processing speed, verbal fluency, cognitive flexibility, planning, creativity, and reasoning. No-training effects were found for far transfer for this group. Far transfer for the logic and planning training group was assessed by measures of processing speed, working memory, verbal fluency, cognitive flexibility, and creativity. Although whether all of those cognitive processes are far transfer for logic and planning training can be debated; nevertheless, no-training effects were found for these tasks for this group either.

Our null findings were not due to lack of motivation, as we measured pre-post motivation for training and found no differences between our two training groups at baseline or over time. Additionally, motivation to train was high (pre-post means above 5.5) on a scale ranging from one (extremely unmotivated) to seven (extremely motivated).

Our null findings fit into a mixed literature on the efficacy of cognitive training to improve cognition in healthy adults. Moreover, meta-analyses reveal conflicting results regarding cognitive gains after training, and study inclusion and analytic techniques have been debated. Three meta-analyses have specifically investigated cognitive training in healthy older adults. Of particular relevance, a meta-analysis of working memory and executive functioning training in older adults (mean age > 60) found that the two types of training did not reliably differ in their ability to change cognition (Karbach and Verhaeghen, 2014). Collapsing across training conditions, there were trend wise effects for near transfer and far transfer. For far transfer, all the individual cognitive domain effect sizes were found to be significant. Of note, the meta-analysis also showed that there were fewer studies with negative results for near transfer effects published than might be expected. This suggests near transfer effects may be over-estimated due to publication bias. Melby-Lervåg and Hulme (2015) criticized this meta-analysis for study inclusion and not using pre-score correction in the statistical analyses. The re-analysis of specific data investigating working memory training and its effects on non-verbal reasoning showed a small overall effect of training when compared to passive controls, and no effect when compared to active controls.

A second meta-analysis in healthy older adults (mean age > 50) found that compared to the active control training condition, cognitive training improved working memory and processing speed, and a composite measure of cognitive function (Kelly et al., 2014). Compared to no intervention, cognitive training improved performance on measures of memory. It is unclear why more change is found when compared to active controls, which is counterintuitive based on the literature. Furthermore, this meta-analysis found training in groups was more effective than individually. A third meta-analysis (mean age ≥ 60) found a small effect for cognitive training compared to active controls, with small to moderate effects for memory, working memory, processing speed, and visuo-spatial skills specifically (Lampit et al., 2014). However, in contrast to the above meta-analyses, this study found no support for cognitive training completed independently at home and no effectiveness for working memory training, which is similar to the present study.

Furthermore, in the area of cognitive training, different statistical analyses have resulted in different results for meta-analyses in younger healthy adults. Focusing on individuals 18–50 years old, a meta-analysis of fluid intelligence gain after dual n-back working memory training reported a small but statistically significant effect of active training compared to control conditions (Au et al., 2015). When Melby-Lervåg and Hulme (2015) reanalyzed the data and accounted for baseline cognitive scores in their effect size calculations, they found smaller effect sizes. Although the effects were significant, the authors argued they were too small to be noteworthy. Furthermore, a re-analysis of the same data using more sophisticated Bayesian analyses did not find support for working memory training related gains when active controls were used; however, it continued to demonstrate effects when passive controls were used (Dougherty et al., 2015). In our study, we also conducted Bayesian analyses and found support for the null hypothesis for cognitive training.

Moreover, complicating the mixed literature, Redick (2015) have questioned positive findings in some studies and revised them to support the null hypothesis. In Redick’s re-evaluation, significant gains in fluid intelligence, originally attributed to working memory training, were due to decreased post-assessment control group scores which resulted in a significant interaction. Of the five studies discussed, one of the studies focused on healthy older adults and the finding of fluid intelligence gain after working memory training was shown to be an erroneous conclusion (Zinke et al., 2014). In the present study, we also found group differences in the overall three group analysis; however, when training groups were compared to the passive control group, we found that differences were due to changes in the performance of the control group, and therefore were not meaningful.

As discussed above, of key theoretical interest is whether cognitive training transfers to fluid intelligence, an idea which is highly contested and debated. In this study, we used working memory training, which is theoretically linked to fluid intelligence, as well as training on higher-level cognitive processes themselves, such as planning, reasoning, and problem solving. In this study, we found that neither training on a cognitive process (i.e., working memory), which is highly correlated with fluid intelligence, nor training on tasks conceptually similar to fluid intelligence, produced gains in Raven’s Progressive Matrices, the most widely used measure of fluid intelligence.

There are a number of potential reasons why we failed to find near and far transfer effects after cognitive training, other than this being a true finding. First, the literature suggests that for cognitive training to be effective, the training has to be challenging but not frustrating (Lövdén et al., 2010). Additionally, participants have to be engaged to push their cognitive limits (Lövdén et al., 2010). Therefore, it could be our cognitive training protocol was not sufficient to meet this goal. However, we chose three different games per training condition to increase the generalizability of the processes trained and prevent boredom or fatigue. The working memory training included an n-back, and two other maintenance and manipulation tasks, one of which had an additional distraction element. The logic and planning training included three games focusing on problem solving, planning, and reasoning. Furthermore, all the games were adaptive and became more challenging as participants improved. Second, it could be the games chosen and/or the amount of training time was not able to keep participants motivated to the level necessary to see gains, despite the improvement on the target measures of training. Third, our lack of findings could be that our healthy participants were already functioning near their cognitive ceiling, which limited their room for growth. In this study, we had strict inclusion criteria to rule out issues that could be associated with cognitive decline, including head trauma, neurological or psychiatric illness, substance use, and health issues.

Although the meta-analyses described above reveal a complex, controversial research area, these studies also suggest possible avenues to reconcile the different findings in the literature. Potential factors that influence the effects of training on transfer include the type of cognitive training, age of the sample, amount of training, presence or absence of randomization, type of control group, geographic location of study, remuneration for participation, and publication type (Melby-Lervåg and Hulme, 2013; Karbach and Verhaeghen, 2014; Au et al., 2015; Melby-Lervåg et al., 2016; Weicker et al., 2016). Additionally, motivation to train and expectations of training related benefits have an effect on training outcomes. Foroughi et al. (2016) manipulated trainee expectancies through their recruitment materials and found that participants who responded to recruitment materials indicating that cognitive training leads to benefits demonstrated an increase on a non-verbal reasoning task after 1 h of training, compared to those that were recruited through neutral materials. Future research on these potential factors will help clarify mixed findings in the training literature.

Limitations of this study include sample size. Although we recruited approximately 30 participants per group, which is larger than many studies in the field, effect sizes for cognitive training studies are generally small; therefore, we were likely underpowered. Nevertheless, well-conducted small studies are still an asset to their field, and can also be entered into meta-analyses. Another limitation of this study included the difficulty in having training programs and cognitive tasks that isolate very specific processes. This is particularly difficult in cognitive training programs assessing working memory and higher-level cognitive processes. The logic and planning training involved working memory processes and the working memory training involved some level of reasoning and learning. However, the extent to which the different processes were emphasized was substantial in the two types of training. Last, we created cognitive composite scores by analyzing correlation patterns between baseline task scores for tasks within a domain. However, the pattern of correlation and relationship between tasks could change after training. Despite these limitations, this study has a number of strengths including using two active training groups plus a passive control, a broad array of near and far transfer measures, and ensuring thorough measurement of demographic, cognitive, health, lifestyle, and other factors that could be related to group differences in cognitive training.

In summary, the cognitive training literature in healthy adults is full of conflicting findings and methodological debates. This study found only practice effects and no near or far transfer after cognitive training in healthy older adults. Whether cognitive training leads to cognitive gains in a consistent manner is yet to be shown, and if gains are to be found they are likely to be small. Future research evaluating mediators and moderators of change will help determine if there are sub-populations of individuals for whom cognitive training may be helpful. Given that better cognitive function in older adults is associated with better physical health and social outcomes (Langlois et al., 2012; Boss et al., 2015; Halil et al., 2015), enhancing cognition will be a topic of continued interest and importance.

## Author Contributions

VG wrote project protocol, supervised data collection and data analysis and wrote the majority of the manuscript. LL-S conducted the analysis and wrote sections of the paper under the supervision of VG. VG and LL-S made an intellectual contribution.

## Conflict of Interest Statement

The authors declare that the research was conducted in the absence of any commercial or financial relationships that could be construed as a potential conflict of interest.

## Footnotes

1 https://www.braingymmer.com/en/
